# Sex differences in the genetic architecture of lifespan in a seed beetle: extreme inbreeding extends male lifespan

**DOI:** 10.1186/1471-2148-9-33

**Published:** 2009-02-06

**Authors:** Trine Bilde, Alexei A Maklakov, Katrine Meisner, Lucia la Guardia, Urban Friberg

**Affiliations:** 1 Animal Ecology/Department of Ecology and Evolution, Evolutionary Biology Centre, Uppsala University, Uppsala SE-753 32, Sweden; 2 Department of Biological Sciences, University of Aarhus, 8000 Aarhus C, Denmark; 3 School of Biological, Earth and Environmental Sciences, The University of New South Wales, Kensington, Sydney, NSW 2052, Australia; 4 Department of Ecology, Evolution and Marine Biology, University of California, Santa Barbara, California 93106-9610, USA

## Abstract

**Background:**

Sex differences in lifespan are ubiquitous throughout the animal kingdom but the causes underlying this phenomenon remain poorly understood. Several explanations based on asymmetrical inheritance patterns (sex chromosomes or mitochondrial DNA) have been proposed, but these ideas have rarely been tested experimentally. Alternatively, sexual dimorphism in lifespan could result from sex-specific selection, caused by fundamental differences in how males and females optimize their fitness by allocating resources into current and future reproduction.

**Results:**

Here we used sex-specific responses to inbreeding to study the genetic architecture of lifespan and mortality rates in *Callosobruchus maculatus*, a seed beetle that shows sexual dimorphism in lifespan. Two independent assays revealed opposing sex-specific responses to inbreeding. The combined data set showed that inbred males live longer than outbred males, while females show the opposite pattern. Both sexes suffered reduced fitness measured as lifetime reproductive success as a result of inbreeding.

**Conclusion:**

No model based on asymmetrical inheritance can explain increased male lifespan in response to inbreeding. Our results are however compatible with models based on sex-specific selection on reproductive strategies. We therefore suggest that sex-specific differences in lifespan in this species primarily result from sexually divergent selection.

## Background

Animals from a broad variety of taxa show sex differences in lifespan, yet a general biological explanation for this phenomenon is lacking [1-6]. Which sex suffers higher mortality varies across species and populations, and is for some species also context-dependent. Nevertheless, some general patterns emerge: in mammals and insects, females commonly live longer than males [e.g. [2,5,7-9]] [but see [10,11]], while males outlive females in most bird taxa [e.g. [4,12,13]].

The sex-specific mortality patterns observed in different taxa have motivated two hypotheses based on genetic asymmetries found between the sexes. The first of these hypotheses, 'the unguarded X' [e.g. [3]], posits that the heterogametic sex should suffer higher mortality rates, since only one copy of the X- (or Z-) chromosome is present there. X- (or Z-) linked recessive deleterious mutations will therefore be unconditionally expressed in the heterogametic sex and cause an overall higher mortality rate. This hypothesis has, as far as we are aware, never been experimentally tested, but is consistent with the reversal of sex-specific mortality rates seen across mammals (heterogametic males) and birds (heterogametic females). The second hypothesis is based on the asymmetrical inheritance pattern of the mitochondria and other cytoplasmic genomes. Since these genomes commonly are only transmitted through females they are consequently only selected in that sex. They may therefore function sub-optimally when expressed in males, which could result in male-biased mortality rates [14]. The fact that several recent studies have indicated that the mitochondria plays an active role in determining lifespan and mortality rates [15-17], coupled with the growing body of research showing that the genome of many species is under different selection pressures in males and females (as shown by sexually differential gene expression [reviewed in [18]] and intra-genomic conflict [19-22]) makes this hypothesis interesting, although it cannot account for the reversal of sex-specific mortality rates seen across taxa.

Another potential explanation for sexual dimorphism in lifespan and mortality rates, is sex-specific selection [2] on resources allocated into current and future reproduction [1,23,24]. Sexually divergent selection on resource allocation may be relatively common, since males and females, in many species, have rather different routes to successful reproduction. Tentative support for this hypothesis comes from comparative studies where sex biases in longevity were attributed to differential investment in reproduction among males and females [4,12], and studies indicating that males from polyandrous species age relatively faster than females compared to males from monandrous species, as predicted by the more intense male-male competition in polyandrous taxa [7,25].

Distinguishing between the various hypotheses for sex differences in lifespan has proven difficult, since comparative and experimental approaches often have given results compatible with several explanatory models [4-6,14,26]. Here we use inbreeding as a tool to investigate sex-specific differences in the genetic architecture of lifespan and mortality rates in the sexually dimorphic seed beetle *C. maculatus *– an emerging model species for studies of quantitative genetics of longevity and ageing [5,8,9,27].

## Results

### Longevity assay I

**Figure 1:**
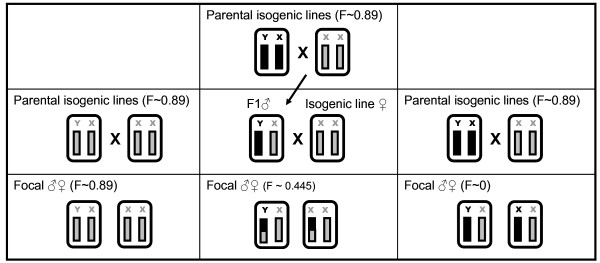
**Schematic design of construction of genotypes**. Triplets of genotypes with three levels of inbreeding were obtained using isogenic lines. Outbred genotypes (F~0) were obtained by crossing two isogenic lines (F~0.89). Male offspring from such crosses were crossed to females from the maternal isogenic line. Offspring from this cross inherited a haploid maternal genome from their mother and a haploid genome from their father, of which half came from the maternal line. Theses individuals were therefore homozygous for approximately 44.5% of their genome. Inbred genotypes came from maternal within-line crosses. With this design, genotypes of three levels of inbreeding produced by each pair of isogenic lines shared the same maternal genome, controlling for maternal effects variation among genotypes within a line-pair.

**Figure 2:**
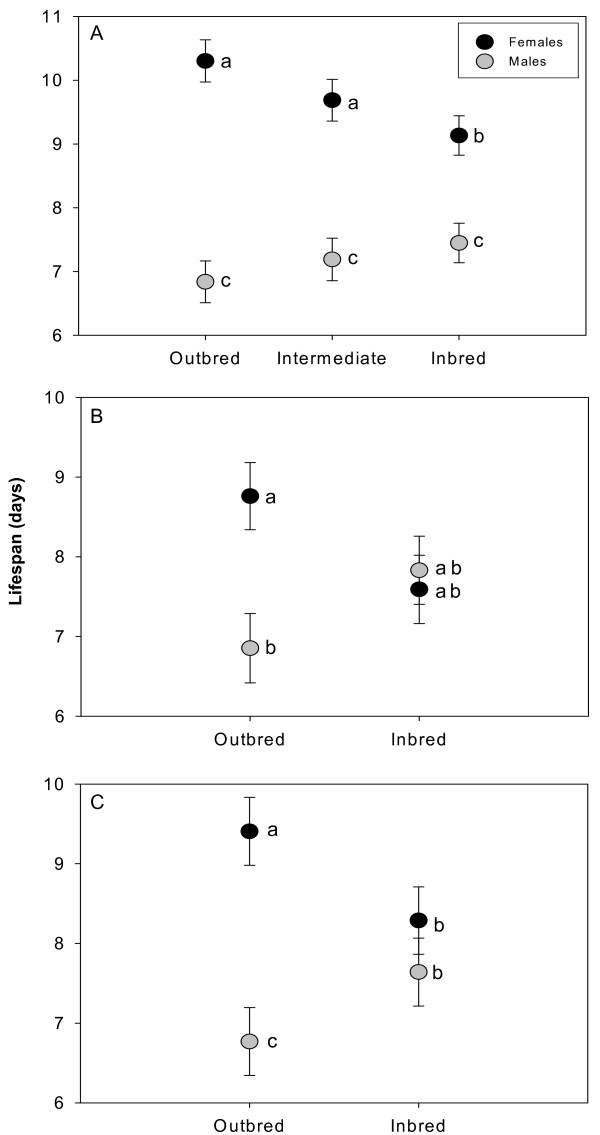
**Sex-specific effect of inbreeding on lifespan in *Callosobruchus maculatus *beetles**. Lifespan (mean days, error bars = SE) of females and males in the longevity assays. (A) Assay I, testing three levels of inbreeding. (B) Assay II, testing two levels of inbreeding, (C) Combined effect of inbreeding (two levels) on lifespan. Significant differences between levels of inbreeding, by contrast analysis, are indicated by different letters.

By pairing 20 distinct isogenic lines we constructed ten experimental genotypes that each formed the basis for three 'sub-genotypes' that varied in level of inbreeding (*F*~0, *F*~0.45 and *F*~0.89) (Fig. 1). Longevity of virgin males and females, from all these genotypes, were then scored in separate assays (see Methods). Sexual dimorphism for lifespan was pronounced across all levels of inbreeding, but no main effect of inbreeding was detected across the sexes for this trait (Fig. 2A, Table 1). The lack of an overall effect of inbreeding was explained by a strong sex × inbreeding interaction (Table 1), indicating that inbreeding had opposing effects on lifespan in males and females (Fig. 2A). Contrast analyses between different inbreeding levels within the sexes did however only show a significant difference between outbred (F~0) and extensively inbred (F~0.89) females (Fig. 2A).

**Table 1:** First assay of sex-specific effects of inbreeding level on lifespan and ageing.

**Source Statistic**	**DF/Variance**	**DFDen/Standard Error**	**F-ratio/Z-value**	**P-value**
**Lifespan**				
Inbreeding	2	49.1	0.66	0.5190
Sex	1	44.6	180.78	< 0.0001
Inbreeding × Sex	2	44.6	7.42	0.0017
Density	1	42.8	5.6	0.0225
Line	0.4232	0.2487	1.7	0.0444
**Baseline mortality (ln α)**				

Inbreeding	2	49.6	0.05	0.9539
Sex	1	44.6	11.47	0.0015
Inbreeding × Sex	2	44.3	2.48	0.0953
Density	1	20.8	0.02	0.8939
Line	0.9632	0.9928	0.97	0.1660
**Rate-of-senescence (β)**				

Inbreeding	2	46.7	0.92	0.4041
Sex	1	42.7	143.37	< 0.0001
Inbreeding × Sex	2	42.6	6.36	0.0038
Density	1	49.1	3.00	0.0894
Baseline mortality (ln α)	1	48.7	332.25	< 0.0001
Late-life deceleration (s)	1	47.2	15.17	0.0003
Line	0.007980	0.004285	1.86	0.0313
**Late-life deceleration (s)**				

Inbreeding	2	46.5	2.75	0.0741
Sex	1	49.8	18.93	< 0.0001
Inbreeding × Sex	2	42.3	3.20	0.0506
Density	1	39.6	0.06	0.8141
Baseline mortality (lnα)	1	50.9	1.78	0.1877
Rate-of-senescence (β)	1	51	13.22	0.0006
Line	0.07719	0.04770	1.62	0.0528

Data was analysed with a General Linear Mixed Model with a REML estimation procedure. Degrees of freedom (DF & DFDen), F-ratios and P-values are provided for fixed effects (Inbreeding, Sex, Inbreeding × Sex and Density); variance components, standard errors, Z-values and P-values are provided for the random factor (maternal line).

**Figure 3:**
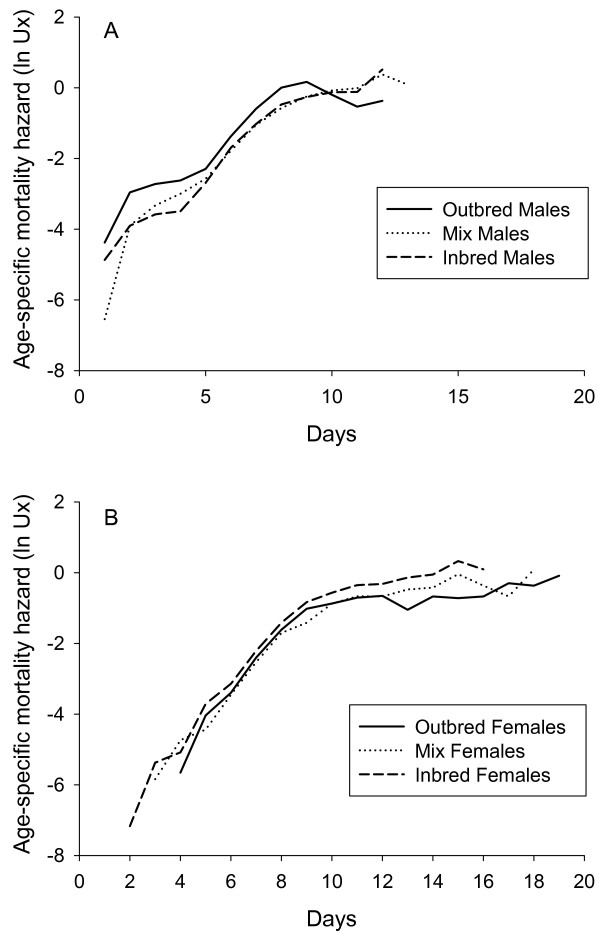
**Age specific mortality curves in *Callosobruchus maculatus *beetles, first assay**. Age-specific mortality hazard (ln Ux) for outbred, intermediate and inbred cohorts of males (A) and females (B) from assay I, using pooled data across lines on daily mortality for each treatment.

To estimate the effect of inbreeding on mortality rates in each sex we fitted different models to the mortality data. The best fit for both sexes was provided by the Logistic model (see Methods), which describes age-specific mortality as a function of three different parameters, baseline mortality (*α*), rate-of-senescence (*β*) and late-life deceleration in mortality (s). The values for the model parameters and their statistical comparison between different levels of inbreeding are presented in Table 2. Age-specific mortality curves are presented in Figure 3. Outbred females had lower baseline mortality and faster late-life deceleration in mortality but higher rate-of-senescence, compared to fully inbred (*F*~0.89) and intermediately inbred (*F*~0.45) females. In contrast, outbred males had higher baseline mortality rate than inbred and intermediately inbred males. Outbred males showed lower rate-of-senescence than fully inbred males, while intermediately inbred males had an intermediate rate-of-senescence, which was not significantly different from the two extremes. There was no effect of inbreeding level on deceleration in late-life mortality in males.

**Table 2:** Sex-specific effects of inbreeding on mortality rates in longevity assay I.

	**Baseline mortality rate ln α (SE)**			**Rate-of-senescence β (SE)**			**Deceleration in late-life s (SE)**		
	F~0.89	F~0.45	F~0	F~0.89	F~0.45	F~0	F~0.89	F~0.45	F~0
**Female**	0.00019a	0.00020a	0.00005b	0.93428a	0.86464a	1.09909b	1.14579a	1.38391a	2.18538b
	(0.00010–0.00034)	(0.00009–0.00047)	(0.00002–0.00012)	(0.85349–1.02273)	(0.75786–0.98646)	(0.98793–1.22276)	(0.93803–1.39956)	(1.05810–1.81005)	(1.83226–2.60656)
**Male**	0.00223a	0.00299a	0.00639b	0.76093a,b	0.70950b,c	0.67197c	0.60176a	0.53157a	0.42345a
	(0.00156–0.00317)	(0.00191–0.00470)	(0.00487–0.00839)	(0.70039–0.82670)	(0.63230–0.79612)	(0.62177–0.72622)	(0.46827–0.77330)	(0.36299–0.77845)	(0.32538–0.55109)

The effect of three levels of inbreeding on mortality and ageing rates in male and female *C. maculatus *beetles. The maximum likelihood estimates of mortality rates (lower and upper confidence intervals) were obtained by fitting the data to a Logistic model and are based on pooled data for each sex and treatment. Letters indicate significant differences in mortality rates between levels of inbreeding within each sex using log-likelihood ratio tests (see text for details).

### Longevity assay II

To test the robustness of the patterns seen in Longevity assay I, we conducted a second experiment contrasting outbred (*F*~0) and inbred (*F*~0.89) genotypes from an independent set of inbred lines, where each level of inbreeding was replicated 11 times. Also this time we found an effect of sex, no main effect of inbreeding and a strong sex × inbreeding interaction (Table 3). There was also a similar trend towards inbred females living shorter and inbred males living longer, as seen in the first assay (Figure 1B).

**Table 3:** Second assay of sex-specific effects of inbreeding level on lifespan and ageing.

**Source Statistic**	**DF/Variance**	**DFDen/Standard Error**	**F-ratio/Z-value**	**P-value**
**Lifespan**				
Inbreeding	1	39	0.03	0.8537
Sex	1	29.8	5.33	0.0281
Inbreeding × Sex	1	29.7	8.84	0.0058
Density	1	29.5	0.00	0.9568
Line	0.2187	0.2770	0.79	0.2149

**Mortality rates, females**				
**Baseline mortality (ln α)**				
Inbreeding	1	14.7	4.87	0.0436
Density	1	18.8	1.98	0.1754
Line	0.4913	0.7436	0.66	0.2544
**Rate-of-senescence (β)**				
Inbreeding	1	16	0.64	0.4353
Density	1	16	0.11	0.7432
Baseline mortality (ln α)	1	14.5	15.44	0.0014
Late-life deceleration (s)	1	16.8	37.73	< 0.0001
Line	0.02133	0.01651	1.29	0.0982
**Late-life deceleration (s)**				
Inbreeding	1	10.9	9.33	0.0111
Density	1	11.3	1.57	0.2359
Baseline mortality (ln α)	1	11.1	8.15	0.0156
Rate-of-senescence (β)	1	11.6	45.45	< 0.0001
Line	0.4392	0.2349	1.87	0.0307

**Mortality rates, males**				
**Baseline mortality (ln α)**				
Inbreeding	1	16.8	2.51	0.1317
Density	1	17	0.18	0.6798
Line	0.2118	0.4854	0.44	0.3313
**Rate-of-senescence (β)**				
Inbreeding	1	16.5	0.04	0.8474
Density	1	17.6	0.51	0.4854
Baseline mortality (ln α)	1	17.3	30.98	< 0.0001
Line	0.002670	0.002788	0.96	0.1691

Data was analysed with a General Linear Mixed Model with a REML estimation procedure. Degrees of freedom (DF & DFDen), F-ratios and P-values are provided for fixed effects (Inbreeding, Sex, Inbreeding × Sex and Density); variance components, standard errors, Z-values and P-values are provided for the random factor (maternal line). The analysis of mortality rates is presented separately for males (Gompertz model) and females (Logistic model) (see text for details).

**Figure 4:**
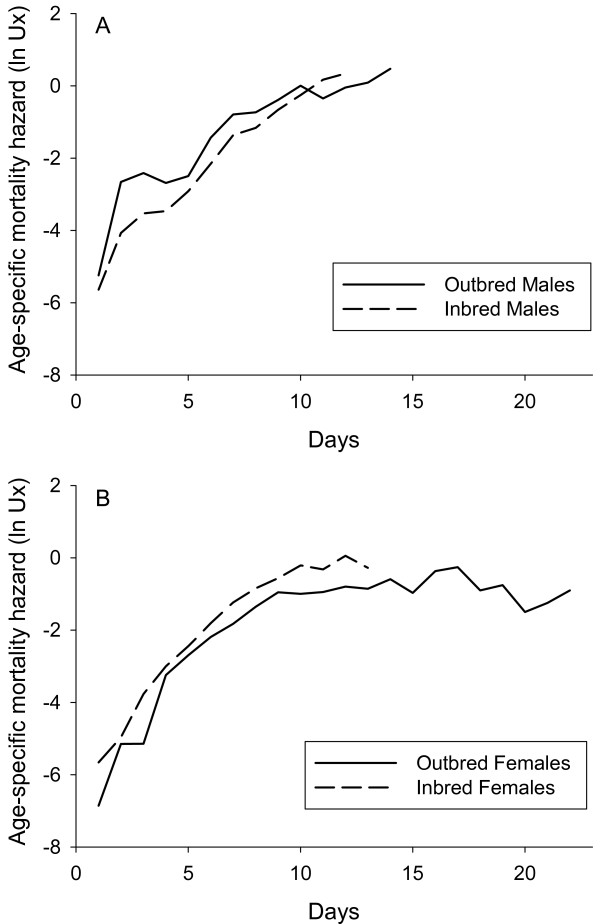
**Age specific mortality curves in *Callosobruchus maculatus *beetles, second assay**. Age-specific mortality hazard (ln Ux) for outbred and inbred cohorts of males (A) and females (B) from assay II, using pooled data across lines on daily mortality for each treatment.

As in the previous assay, there was a difference in the effect of inbreeding on mortality rates estimated from the Logistic model (see Methods). Outbred females again had lower baseline mortality and faster late-life deceleration than inbred females (Table 4), while there in this assay was no difference in the rate-of-senescence between inbred and outbred females. Outbred males again had higher baseline mortality rate than inbred males, but there was no difference in the two other model parameters between outbred and inbred males.  Age-specific mortality curves are presented in Figure 4.

**Table 4:** Sex-specific effects of inbreeding on mortality rates in longevity assay II.

	**Baseline mortality rate α (SE)**		**Rate-of-senescence β (SE)**		**Deceleration in late-life s (SE)**	
	F~0.89	F~0	F~0.89	F~0	F~0.89	F~0
**Female**	0.00241a	0.00134b	0.73184a	0.77611a	0.79646a	1.72506b
	(0.00164–0.00355)	(0.00093–0.00194)	(0.66356–0.80713)	(0.71210–0.84558)	(0.61250–1.03567)	(1.47576–2.01649)
**Male**	0.00392a	0.01220b	0.57590a	0.52531a	0.25954a	0.39520a
	(0.00283–0.00545)	(0.01005–0.01480)	(0.52340–0.63368)	(0.48552–0.56835)	(0.14724–0.45748)	(0.29585–0.52792)

The effect of two levels of inbreeding on mortality rates in male and female *C. maculatus *beetles. The maximum likelihood estimates of mortality rates (lower and upper confidence intervals) were obtained by fitting the data to a Logistic model and are based on pooled data for each sex and treatment. Letters indicate significant differences in mortality rates between levels of inbreeding within each sex using log-likelihood ratio tests (see text for details).

### Combining the longevity data

To conduct a more powerful test of the similar trends we found in the two longevity assays we combined the data from the two assays (but excluded the intermediately inbred genotypes that only was present in assay I), and treated the two assays as independent blocks. This analysis also showed an effect of sex, no general inbreeding effect and a strong sex × inbreeding interaction (Table 5). Contrasts from this analysis confirmed the trend seen in the two assays, and show that inbred females lived significantly shorter than outbred females and that inbred males lived significantly longer then outbred males (Fig. 1C).

**Table 5:** Sex-specific effects of inbreeding on lifespan on combined data from assays I & II.

**Source Statistic**	**DF/Variance**	**DFDen/Standard Error**	**F-ratio/Z-value**	**P-value**
Inbreeding	1	72.9	0.18	0.6685
Sex	1	59.8	42.26	< 0.0001
Inbreeding × Sex	1	59.9	15.51	0.0002
Density	1	25	0.28	0.5987
Line	0.2198	0.1913	1.15	0.1253
Block	0.2032	0.4146	0.59	0.3120

Data was analysed with a General Linear Mixed Model with a REML estimation procedure. Degrees of freedom (DF & DFDen), F-ratios and P-values are provided for fixed effects (Inbreeding, Sex, Inbreeding × Sex and Density); variance components, standard errors, Z-values and P-values are provided for the random factor (maternal line).

### Fitness assay

We tested if inbreeding was associated with a reduction in fitness for both males and females by conducting fitness assays, where lifetime offspring production were recorded independently for each sex using the genotypes from the second longevity assay (see Methods). As expected, we found that inbreeding depressed fitness significantly in both sexes (Table 6, Weighted Matched Pairs test, females: t_10 _= 10.77, *P *< 0.0001; males, t_10 _= 5.77, *P *= 0.0002).

**Table 6:** Sex-specific effects of inbreeding on fitness (lifetime adult offspring production).

**Sex**	**Inbred F~0.89**	**Outbred F~0**	**Inbred/Outbred × 100**
Female	30.96 (3.77)	89.02 (5.04)	35%
Male	42.60 (5.59)	90.50 (6.80)	47%

Fitness assays of male and female *C. maculatus *beetles, data show lifetime adult offspring production (mean ± SE) and the relative value of inbred to outbred reproductive success in %.

## Discussion

Our finding that outbred females lived longer than outbred males is consistent with results from previous studies of *C. maculatus *[5,8,27,28], as is the finding that inbred females live shorter than outbred females [5]. In contrast, the results showing that inbred males live longer than outbred males is noteworthy and has no prior empirical parallel. Below we first address how this result relates to earlier studies on the genetic architecture of lifespan, and then discuss our findings in relation to theories for sexual dimorphism in lifespan.

In a previous study, using this species and strain, Fox et al. (2006) [5] found that male lifespan did not change when males were inbred to a moderate level (F~0.25). Our data, on the other hand, suggest that there is a positive linear relationship between inbreeding level and male lifespan, and show that male lifespan is extended by inbreeding when comparing outbred and extensively inbred (F~0.89) males across assays. However, we could not detect a significant difference in lifespan between males that were outbred and those that were intermediately inbred (F~0.44), and to that extent our results are not inconsistent with the ones obtained previously. One possible explanation as to why inbreeding had no positive effect on lifespan in the study by Fox et al. (2006) [5] is that they used a different experimental protocol. In our experiments, virgin beetles were kept in same-sex groups, while virgin beetles were kept in isolation in the study by Fox et al. (2006). Virgin males live considerably longer when kept in isolation, compared to when kept in same-sex groups or when males are kept with females (A.A. Maklakov and R. Bonduriansky, unpublished data), indicating that that male behaviour is context-dependent. Males of this species normally spend much of their time chasing potential mates and trying to copulate with them, which they are not stimulated to do when kept alone. They do however perform these behaviours when kept in same-sex groups. The intrinsic difference in how inbred and outbred males spend their energy may not be realized if they do not meet conspecifics, which may explain why inbred males did not have prolonged lifespan in the study by Fox et al. (2006) [5]. Another difference in the experimental approaches is that Fox et al. 2006 [5] used one generation of full-sib matings whereas we used ten, which could have resulted in more opportunity for purging of deleterious mutations in our lines. An indication that this did affect our results is that we could not detect a significant difference in lifespan between outbred and intermediately inbred (F~0.44) females, while Fox et al. (2006) found such difference when comparing outbred and less inbred females (F~0.25). It is not obvious how purging could have accounted for the increased male lifespan with inbreeding observed in our study, given that the potential effect of purging did not prevent fitness of inbred individuals of both sexes to be severely reduced.

### Asymmetric inheritance

As outlined in the introduction, sex-biased mortality rates can theoretically be caused by unconditional expression of recessive deleterious mutations on the X/Z chromosome in the heterogametic sex, and by cytoplasmic genomes that not are optimized for functioning in males. A distinct prediction made by 'the unguarded X'-hypothesis is that the heterogametic sex (males in *C. maculatus*) should suffer less from inbreeding compared to the homogametic sex (i.e. females here), since the heterogametic sex will not suffer from increased expression of X/Z-linked recessive deleterious mutations [4,5,14]. In our assays males did suffer less than females, in terms of reduced lifespan, in accordance with this theory. However, given that we noted a significant shift towards longer male lifespan with inbreeding, we conclude that this model cannot adequately explain the observed patterns. Likewise we conclude that the observed responses to inbreeding can not be explained by the mitochondrial, or any other cytoplasmic genome, since these always are haploid and therefore are unaffected by inbreeding. A cytoplasmic effect was also ruled out in a previous experiment with this species, using inter-population crosses [27]. We do note however that our results do not exclude the possibility that the magnitude of sexual dimorphism for lifespan in *C. maculatus *could be influenced by the X-chromosome or cytoplasmic genomes.

### Sex-specific selection

Sex-specific selection offers an alternative explanation as to why the sexes display differences in mortality rates and longevity, and presents a range of possible mechanisms through which prolonged lifespan of inbred males can occur. These arguments are all based on the assumption that the optimal reproductive strategies of males and females differ, which involves sex-specific investments in current and future reproduction [6,25,29-31]. Below we discuss several possible scenarios of how sex-specific selection on the genetic architecture of lifespan could have caused our results.

Many types of behaviour required for successful male reproduction (e.g. mate search, male-male competition and courtship) are associated with costs. For males of *C. maculatus*, these costs of reproduction cause reduced longevity [32]. It has further been shown that the cost of living in same-sex groups is equal to the cost of reproduction for males of this species (A. A. Maklakov & R. Bonduriansky, unpublished data), which indicates that males exhibit a similar behavioural repertoire in same-sex groups, as when exposed to females. If inbred males are less likely to engage in energy-demanding reproductive behaviours, either because their impaired phenotypic condition prevents them or because of condition-based strategic decisions [30], the result could be both prolonged lifespan and reduced fitness, as observed here. Hence, males should adopt a 'live fast, die young' strategy [6]. In contrast, females do not engage in many of the costly male behaviours and are instead predicted to optimize their reproductive output by conserving energy for oviposition, provisioning of offspring, foraging and predator avoidance. Inbred females might therefore be expected to experience reduction in both fitness and lifespan consistent with the observed data. This explanation may be particularly relevant to the species studied here, since adult *C. maculatus *normally have no access to food and water [27], which causes used resources to be inversely related to resources left to consume.

When a trait is under sex-specific selection, it is constrained to evolve to its sex-specific optima, because the sexes share the same genome. Eventually, such intra-locus sexual conflicts are thought to be resolved by genes evolving to become sex-limited in expression [33-37]. This process is however inherently slow [34,36,38], which is why many sexually dimorphic traits currently should experience opposing directional selection in the two sexes. Directional selection typically results in directional dominance (i.e. the selected trait shows dominance in the direction of the preferred phenotype) [39,40] and sex-limited genes coding for traits under sexually divergent selection are therefore predicted to show directional dominance in opposite directions in males and females. Hence, if resource allocation into current and future reproduction is under opposing directional selection in males and females, and a subset of the genes coding for this trait is sex-limited in expression, inbreeding could decrease female lifespan while increasing male lifespan. This possibility is also partially supported by a previous study on this species, where it was shown in population crosses that genes coding for prolonged lifespan were generally dominant in females, while this pattern was much weaker in males [27].

Finally, there is also the possibility that substantial autosomal intra-locus sexually antagonistic genetic variation exists for allocation of resources into current versus future reproduction. Although theory have shown that such variation is not easily maintained on the autosomes [41], the possibility that the dominance/recessive patterns are reversed in the sexes have yet not been explored. Intuitively, stable polymorphisms could be maintained on the autosomes if male beneficial allelic versions were dominant in males and recessive in females, while female beneficial allelic variants were dominant in females and recessive in males. Inbreeding would then increase the homozygosity of such genes and move the trait value in the opposite, and non-preferred, direction in both sexes.

## Conclusion

We tested how inbreeding affects lifespan in *C. maculatus*, a seed beetle which is sexually dimorphic for this trait. Our results show that extensive inbreeding shortens female lifespan, while it extends male lifespan. Fitness was however substantially, and similarly, depressed by inbreeding in both sexes. These results show that the genetic architecture of lifespan is different in males and females, but the data are not consistent with the predictions made by any model based on asymmetrical inheritance patterns. Our results are consistent with models based on sex-specific selection on reproductive strategies, such that males, in contrast to females, maximize fitness by early and intense investment in reproduction traded off against shorter lifespan [6,7,25,30,42,43].

## Methods

### Study animal

We used a South-Indian strain of the seed beetle *Callosobruchus maculatus*, obtained from Prof. C. W. Fox, University of Kentucky. This population originates from a few hundred infested beans (mung, *Vigna radiate *and black gram *V. mungo*) collected in 1979 [44]. In the lab adults are kept on mung beans as egg laying substrate. When the larva hatches, it burrows into the bean where it feeds, grows and pupates, with a generation time of approximately 24 days at 25°C. The beetles only feed during their larval stage and no water or other resources are obtained by the adults. During the last ~200 generations our lab population has been maintained at > 1000 adult individuals per generation [45], and the beetles have thus had a long period to adapt to the laboratory environment. We kept the beetles at 25°C, 60% relative humidity and 14:10 light:dark cycle in climate chambers.

### Construction of isogenic lines

Isogenic lines were founded in January 2005. Two-hundred and fifteen virgin females were mated once and placed individually in 30 ml plastic vials with > 100 mung beans. We isolated beans with single hatched larvae prior to adult eclosion. Offspring from each of these females were then used to construct isogenic lines through controlled full sib matings for ten consecutive generations. To reduce the loss of lines during the inbreeding process, two pairs of full-sibs were mated in parallel every generation (only offspring from one pair was retained). Despite this effort only about 40% of the lines survived. After ten generations of full sib matings 89% of the genome is expected to be homozygous, when conservatively assuming that individuals used in the first cross all where heterozygous at all loci and that the males and females initially crossed carried different alleles at all loci [46]. The lines were then propagated and maintained as populations at more than 100 individuals.

### Experimental genotypes

We selected 20 isogenic lines to obtain experimental genotypes of three different inbreeding coefficients (outbred *F*~0; intermediately inbred *F*~0.45; and inbred *F*~0.89, Figure 1). To accomplish this we divided the 20 isogenic lines into 10 pairs, of which one in each pair was randomly assigned to be the maternal line. By three different crossing designs, we then constructed individuals with the three inbreeding coefficients, from each pair of inbred lines. Inbred genotypes were offspring from maternal within-line matings, and outbred individuals were offspring from crossing females from the maternal lines to males from their paired paternal line. To obtain genotypes of intermediate level of inbreeding we first mated females from the maternal line to males from their paired paternal line (1^st ^generation in Figure 1). Male offspring from these crosses were then mated to females from their maternal line (2^nd ^generation in Figure 1). Offspring from the last cross inherited a haploid maternal genome from their mother, and a haploid genome from their father of which half came from the maternal line. These individuals were therefore homozygous for 44.5% of their genome (3^rd ^generation in Figure 1). Using this design, genotypes of three levels of inbreeding produced by each pair of isogenic lines shared the same maternal genome. We were thus able to control for maternal effects variation among genotypes within a line-pair, which previously have been shown to have a significant effect on lifespan and fitness in this species [27,47]. Within each line-pair, beetles of each of the three genotypes were propagated in two different jars to diminish effects by a shared environment.

### Longevity assays

Individual beans with a single egg cemented onto their surface were isolated until hatching. On the day of eclosion, virgin adult beetles were transferred to Petri dishes holding 50 ml of mung beans, with other beetles from the same Line, Inbreeding Level and Sex. The Petri dishes were then scored daily for dead beetles. As eclosion of beetles was not completely synchronous, new Petri dishes were started daily. The density of beetles per Petri dish therefore varied between days (mean ± SE: 21.33 ± 13.57). Beetles were maintained at similar environmental conditions as described above. Overall we scored 3132 females and 3117 males for the age at death.

To confirm our original findings we conducted a second experiment. The general protocol was the same as in the first experiment except for exclusion of the intermediate level of inbreeding, i.e. we contrasted sex-specific longevity and mortality rates of inbred (*F*~0.89) and outbred (*F*~0) individuals. This time we used 11 different pairs of isogenic lines to create 11 pairs of inbred and outbred genotypes sharing the maternal genome. In this experiment we scored 3056 females and 3019 males for the age at death.

A potential problem associated with assaying mortality rates of beetles housed in groups is that estimates of mortality rates can be influenced by densities declining at different rates among genotypes, as individuals die off. However, if anything, this should result in conservative estimates of differences in lifespan between genotypes, since short lived genotypes on average will be less stressed and affected by other individuals, and therefore should live relatively longer than if the densities were kept constant. We also accounted for density in our statistical models (see below).

### Statistical analyses of longevity and mortality rates

We used the mixed general linear model with REML estimation procedure to test for the effect of Sex, Inbreeding Level and Sex × Inbreeding Level on lifespan and the different model parameters that describe the mortality curve, in each assay separately. We treated Sex and Inbreeding Level and their interactions as fixed effects, while maternal line was treated as a random factor. Density was used as a covariate in all models. The data on longevity and mortality rates were averaged across jars and Petri dishes for each sex and genotype. We used within-model contrasts to compare the different levels of inbreeding within each sex. To increase power we also analyzed the two assays jointly, using only the data on outbred (*F*~0) and fully inbred (*F*~0.89) beetles. In this combined analysis, assay (I and II) was modeled as a random Block effect. We tested for Block × Sex and Block × Inbreeding Level interactions, which were not significant and were subsequently removed from the model.

To analyse the effect of Inbreeding Level on age-specific mortality we pooled our data across lines from each sex and treatment to increase the resolution of the analyses, because small sample sizes provide very inaccurate estimates of mortality rates [48]. This analysis did not allow using density as a covariate. Therefore, we first tested for the effect of density on mortality rates using the same mixed model approach as for longevity data (see above). There was no significant effect of density on any mortality rate estimate in neither assay (Tables 1 &2). We used maximum likelihood (ML) estimation (WinModest software package [49]) to compare four models that describe the demographic rate of change in mortality with age: Gompertz, Gompertz-Makeham, Logistic and Logistic-Makeham. We used Likelihood ratio tests to find the model that gave the best fit to our data. The best fit for both sexes was provided by the Logistic model, *μ*_*x *_= *α e*^*βx*^/[1 + (*αs*/*β*)(*e*^*βx *^- 1)], where *μ*_*x *_is the mortality hazard at age *x*, *α *is the baseline mortality rate, and *β *is the rate of increase in mortality with age (i.e. the rate-of-senescence), and *s *is the rate of deceleration late in life [49]. We note that for two Inbreeding levels in the first assay, the best fit for male cohorts was provided by the Logistic-Makeham model, but we chose to use Logistic model in all of our analyses for consistency. We used likelihood ratio tests to statistically compare the model estimates for each sex among different Inbreeding levels within each assay. For example, in order to test for the difference between the baseline mortality rate, *α*, in inbred and outbred females in the first assay, we calculated the difference between log-likelihoods of the null hypothesis model (L-L_null_) where *α *was constrained and the alternative model (L-L_alt_) where all three model parameters (*α*, *β *and s) could vary [49]. We then compared -2(L-L_null _- L-L_alt_) to a chi-square distribution with one degree of freedom (equal to the number of constrained parameters).

### Fitness assay

Fitness assays were conducted of the inbred and outbred genotypes that were used in the second longevity assay. We determined lifetime offspring production for females and males in separate assays. Fitness assays were conducted in small populations (N = 20), simulating semi-natural conditions. In the female assays, three replicates of five females from each genotype were sampled and each placed in a 60 mm Petri dish with excess mung beans for oviposition along with five sterile base population females and ten virgin base population males. In the male assays, three replicates of five males from each genotype were kept with five sterile base population males and ten virgin base population females. For each sex a total of 132 Petri dishes were assayed (11 replicated inbred and 11 outbred genotypes, each propagated from 2 different jars, from which 3 replicates were created = 132). Sterilization of beetles was done by irradiation with a dose rate of 2.16 Gray per minute for a 32 minute period (inherent filtration 2.2 mm Cu) at Aarhus University Hospital. The dose was set to 70 Gray based on previous experiments with this species [50]. Irradiation did not affect female fecundity but resulted in the production of sterile eggs, nor did it affect male insemination ability while ensuring sterility of sperm. From each replicate, egg laiden beans were collected and kept under experimental conditions (as above) until all offspring eclosed. The total number of adult offspring produced from each replicate was scored and taken to represent fitness. The effect of inbreeding level on reproductive success was analyzed separately for each sex using Weighted Matched Pairs tests.

## Authors' contributions

TB, AAM and UF designed the experiments. TB, KM and LG performed the experiment. AAM and TB analysed the data and TB, AAM and UF wrote the paper. The authors read and approved the final manuscript.
